# Identification and Classification of Hubs in Brain Networks

**DOI:** 10.1371/journal.pone.0001049

**Published:** 2007-10-17

**Authors:** Olaf Sporns, Christopher J. Honey, Rolf Kötter

**Affiliations:** 1 Department of Psychological and Brain Sciences and Program in Cognitive Science, Indiana University, Bloomington, Indiana, United States of America; 2 Department of Cognitive Neuroscience, Section Neurophysiology & Neuroinformatics, Radboud University Medical Center, Nijmegen, The Netherlands; 3 C. & O. Vogt Brain Research Institute and Institute of Anatomy II, Heinrich Heine University, Düsseldorf, Germany; University of Newcastle, United Kingdom

## Abstract

Brain regions in the mammalian cerebral cortex are linked by a complex network of fiber bundles. These inter-regional networks have previously been analyzed in terms of their node degree, structural motif, path length and clustering coefficient distributions. In this paper we focus on the identification and classification of hub regions, which are thought to play pivotal roles in the coordination of information flow. We identify hubs and characterize their network contributions by examining motif fingerprints and centrality indices for all regions within the cerebral cortices of both the cat and the macaque. Motif fingerprints capture the statistics of local connection patterns, while measures of centrality identify regions that lie on many of the shortest paths between parts of the network. Within both cat and macaque networks, we find that a combination of degree, motif participation, betweenness centrality and closeness centrality allows for reliable identification of hub regions, many of which have previously been functionally classified as polysensory or multimodal. We then classify hubs as either provincial (intra-cluster) hubs or connector (inter-cluster) hubs, and proceed to show that lesioning hubs of each type from the network produces opposite effects on the small-world index. Our study presents an approach to the identification and classification of putative hub regions in brain networks on the basis of multiple network attributes and charts potential links between the structural embedding of such regions and their functional roles.

## Introduction

Large-scale cortical networks, comprising anatomically distinct regions and inter-regional pathways [1]–[3], exhibit specific non-random connection patterns [4]. The structural (i.e. topological) features of large-scale cortical networks are of special interest as they may be linked to aspects of brain function. Structural analyses have utilized a wide spectrum of graph theoretic measures [5], [6] including clustering coefficients and the distributions of node degrees, path lengths and structural motifs. Brain networks have been found to exhibit high levels of clustering combined with short average path lengths, a pattern indicative of a small-world architecture [7]–[11]. It has further been argued that the structural characteristics of brain networks contribute to their functional organization by promoting functional segregation and integration [12], [13], high neural complexity [8], [14], the minimization of processing steps [15], efficient wiring [16] and synchronizability [17].

Global structural parameters can reveal the organization of an entire network, but they cannot capture the contributions of individual network elements (e.g. brain regions). The manner in which individual brain regions are embedded within the overall processing architecture may determine how they participate within the dynamics of the network. Passingham et al. [18] formulated the hypothesis that the connectional fingerprint of a brain area (i.e. its specific pattern of efferent and afferent connections within the network) might define its functional role. Network participation indices capturing some local statistics of degree distributions (density, transmission, and symmetry; [19]) revealed significant differences across brain regions in macaque cortex and highlighted the relations between their individual topological and functional characteristics. An analysis of the contributions of individual brain regions to the global distribution of structural motifs within macaque visual cortex identified significant differences among individual brain regions [20]. Several EEG, MEG and fMRI studies have collected functional network indices of individual brain regions [21], [22], revealing changes in regional network indices in response to experimental perturbation [22], [23]. In recent work with a large-scale cortical model [24] we observed that structurally central brain regions tended also to have elevated centrality within corresponding functional networks. Some hub regions appeared to link multiple functional clusters (e.g. visual and sensorimotor) while others occupied central positions within a single functional cluster.

In this paper we aim to more fully characterize the structural embedding of both types of hub regions in brain networks and to determine whether our results can be extended beyond the brain of a single species. Examining large-scale connection matrices for macaque and cat cortex we focus on structural motif distributions and centrality measures of vertices with high degree because of their potential for relating local processing characteristics to global functional interactions and robustness in these networks. Motifs are classes of subgraphs from which larger networks can be composed [25], [26]. Centrality measures, in general, capture the structural importance of a vertex with respect to the rest of the network [27]. While hubs are often identified solely on the basis of their high degree, the relationships between degree, motif contributions as well as betweenness centrality and closeness centrality of individual brain regions have not previously been investigated in detail. We show that the intersection of node degree, motif fingerprint, betweenness and closeness allows the identification of hub regions, many of which have previously been classified as polysensory or multimodal. We then classify these hub regions into provincial and connector hubs [22], [28], a distinction that is based on whether they tend to link other vertices within a single module or whether they link different modules to one another. We show that lesioning of provincial hubs decreases the small world index while lesioning connector hubs produces the opposite effect.

## Methods

### Data Sets

**Figure 1:**
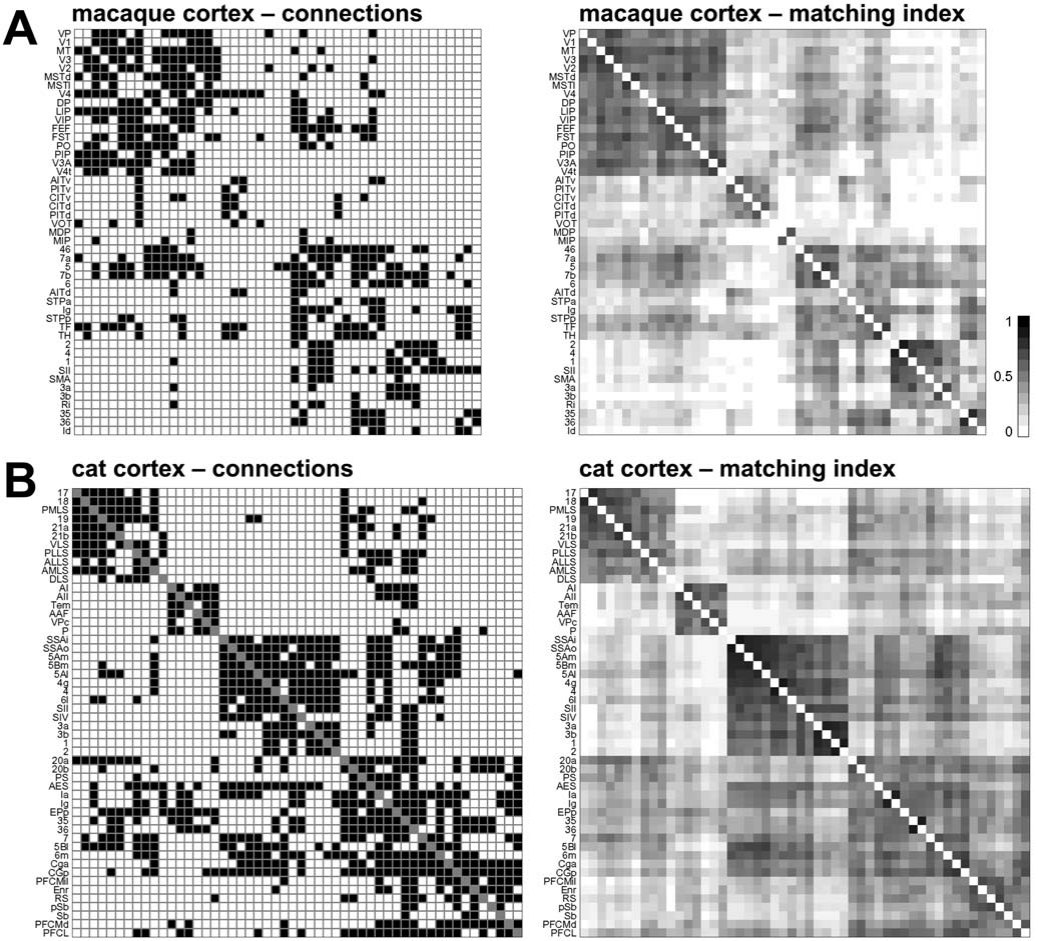
Connection matrices and matching index matrices for data sets examined in this study. Plots show structural connections (left panels) and matching index (right panels). Connection patterns are represented as binary connection matrices C_ij_, with existing connections (edges) indicated by a filled (black) square (c_ij_ = 1). No distinction is made between connections that have been shown to be absent and connections that are unknown; all are represented by a white square (c_ij_ = 0). Main diagonals are indicated in grey and self-connections are excluded (c_ii_ = 0). From top to bottom: (A) Macaque cortex (*N = *47, *K = *505). (B) Cat cortex (*N = *52, *K = *820). Panels on the right show the matching index matrix M_ij_ calculated from the connection matrix following Hilgetag et al. [6]. The matching index scales between 0 (no match) and 1 (perfect match), and m_ij_ = m_ji_. The arrangement of brain regions for each of the four matrices was arrived at as follows. The M_ij_ matrix was converted to a distance matrix, from which a hierarchical cluster tree was computed using a consecutive linking procedure based on farthest inter-cluster distances. This resulted in a linear ordering of areas based on cluster membership and inter-cluster distances. The ordering was rotated such that visual areas appear topmost.

We examine two data sets, to be referred to in this paper as “macaque cortex“ and “cat cortex”. Macaque cortex and cat cortex contain predominantly isocortical brain regions. All data sets consist of binary connection matrices of brain regions connected by inter-regional pathways. Diagrams of the connection matrices are shown in Figure 1, area names and abbreviations are provides in the Supporting Information (Text S1). Data sets can be also be downloaded at http://www.indiana.edu/∼cortex/connectivity.html.

Macaque cortex is an updated network matrix generated following the parcellation scheme of Felleman and Van Essen [1], including visual, somatosensory and motor cortical regions as well as their interconnections [24]. The data were manually collated in the CoCoMac database from published tracing studies according to standard procedures [29], [30]. Subsequently, all relevant data were translated algorithmically to the Felleman and Van Essen map using coordinate-independent mapping [9], [31]. Following resolution of redundant and inconsistent results a binary connection matrix with *N* = 47 and *K* = 505 was generated. To estimate projection lengths we calculated distances between center-of-mass coordinates for each connected pair of brain regions using the Caret macaque cortex surface map (http://brainmap.wustl.edu/caret; [32]), as previously described [15].

Cat cortex is derived from the matrix published by Scannell et al. [2]. We discarded area Hipp (hippocampus) and all thalamic regions and thalamo-cortical pathways. The resulting matrix was converted to binary format and has *N = *52 and *K = *818.

### Graph Theory Methods

Graphs are composed of vertices (or nodes, here equivalent to brain regions) and edges (or connections, here equivalent to inter-regional pathways). The connectivity structure of a graph is represented by its adjacency matrix, here an asymmetric binary matrix representing directed but unweighted edges. Paths are ordered sequences of edges linking pairs of vertices (a source and a target). The distance between two vertices corresponds to the length (number of edges) of the shortest path between them. The distance matrix of a graph comprises all pair-wise distances. Its maximum corresponds to the graph diameter, its minimum to the graph radius, and its average to the graph's characteristic path length.

Basic graph measures such as connection density, proportion of reciprocal connections, degree distributions, measures derived from the distance matrix (diameter, radius, path length), and clustering coefficients were calculated using standard graph theory methods, reviewed in detail elsewhere ([5]; a Matlab (Mathworks, Natick, MA) toolbox as well as other files related to this paper can be downloaded at http://www.indiana.edu/∼cortex/connectivity.html).

Network topology may be said to correspond to a “small world” [33] if the network's clustering coefficient is much greater than that of equivalent random controls *γ>>γ_random_*, while their path lengths are comparable *λ≈λ_random_*. The small-world index *σ_sw_*, introduced by Humphries et al [34], is defined as:
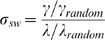



Comparisons are carried out against populations of n = 1000 degree-matched random networks (see below).

### Motif Detection

Structural motifs (or subgraphs) of size *M* consist of *M* vertices and a set of edges (maximally *M^2^−M*, for directed graphs, minimally *M−1* with connectedness ensured). For each motif size *M* there is a limited set of distinct motif classes. For example, there are 13 motif classes for motif size *M* = 3. A Matlab toolbox for detecting and counting motifs of sizes 2*≤M≤*5 is available at http://www.indiana.edu/∼cortex/connectivity.html.

When assessing motif contributions (cf. [20]), we carried out a dual comparison to two different random models that jointly control for the effect of degree sequences and potential neighborhood relations. Two populations of control networks (both with *n = *100 exemplars) were constructed, using a Markov switching algorithm that preserves degree sequences [35]. The first population of controls, the ‘Random’ (randomized) networks, preserved the number of network vertices and edges as well as their degree sequences. For each random network, 2×10^6 ^switches were carried out. The second population of controls, ‘Lattice’ (latticized) networks, were constructed like random networks, but in addition edges are redistributed such that they lie close to the main diagonal of the connection matrix (after an initial random permutation of the vertices). This approach tends to generate randomized networks that incorporate nearest-neighbor connectivity as found in a ring or lattice topology. Thus, lattice networks incorporate a variant of local aggregation or neighborhood relations between vertices, a feature not captured by the random null hypothesis [36]. Motif counts were considered statistically significant if z-scores exceeded+2, +3, or higher values for comparisons to both random controls as well as lattice control networks (*n = *100).

### Centrality Measures

Central vertices in a network are those that have structural or functional importance, for example by serving as waystations for network traffic (analogous to bridges or connectors) or by influencing many other vertices through short and direct paths. Several concepts and measures of centrality have been proposed [27] that capture the degree of “betweenness” [37] or “closeness” [38] of a vertex within the overall network architecture. The closeness centrality of vertex *i* is calculated as the inverse of the average distance from this vertex to all other vertices in the network (i.e. the inverse of the row mean of the distance matrix):
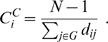



The definition is suitable if the graph G is fully connected, as is the case for all data sets considered in this study. Note that our definition of closeness centrality uses the lengths of all outgoing shortest paths starting from a central vertex; other definitions of closeness centrality are possible based on the lengths of incoming shortest paths (“in-closeness centrality”), or all distances. It is also worth noting that closeness centrality is directly proportional (scaled up by a factor N(N-1)^2^) to the local “efficiency” that was later defined in [39] and employed in the analysis of functional brain networks [23].

The betweenness centrality of a vertex is here defined as the fraction of shortest paths between any pair of vertices that travel through the vertex [37]. The betweenness centrality of a vertex *i* is given as
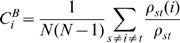
where *ρ_st_*(*i*) is the total number of shortest paths between a source vertex *s* and a target vertex *t* that pass through *i*, and *ρ_st_* is the total number of all shortest paths linking *s* to *t*. To calculate betweenness centrality we applied an efficient Matlab algorithm developed by Gleich [40].

### Community Structure and Hub Classification

To identify modules (communities) within each network, we apply a variant of a spectral community detection algorithm [41]. As inputs to the algorithm we used matrices of matching indices [6], which express the similarity of connection patterns for each pair of vertices (Figure 1). Once modules were detected, different solutions were ranked according to a cost function and the optimal modularity (out of 10000 solutions for a range of between 2 and 6 modules) was used as the basis for hub classification. To classify hubs we calculated each vertex's participation index *P* [28], [42], which expresses its distribution of intra- versus intra-module connections. *P* of vertex *i* is defined as
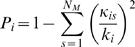
where *N_M_* is the number of identified modules, *k_i_* is the degree of node *i*, and κ_is_ is the number of edges from the *i*th node to nodes within module *s*.

Considering only high-degree vertices (i.e. vertices with a degree at least one standard deviation above the network mean) we classify vertices with a participation coefficient *P*<0.3 as provincial hubs, and nodes with P>0.3 as connector hubs. Since *P* cannot exceed 0.5 for two-module networks and 0.67 for three-module networks, kinless hubs (i.e. nodes with P>0.8 [32c]) cannot occur in these mammalian cortical networks.

## Results

We calculated network measures and motif distributions for two mammalian connectivity data sets, macaque cortex and cat cortex (see Methods). The connectivity data sets are shown in Figure 1, with brain areas arranged according to a cluster analysis based on the matching indices [6] for all area pairs. The matching index quantifies the overlap in afferent and efferent connections between two areas, and previous studies have suggested that areas with low pair-wise similarity in their patterns of afferents and efferents tend to have different functional properties [18]. The matching index matrix, when subjected to cluster analysis, then serves to group areas. For macaque and cat cortex, the resulting arrangement of areas resembles the major functional subdivisions (e.g. visual, sensorimotor, auditory, prefrontal) of mammalian cerebral cortex, confirming that groups of functionally related areas share connection patterns.

**Figure 2:**
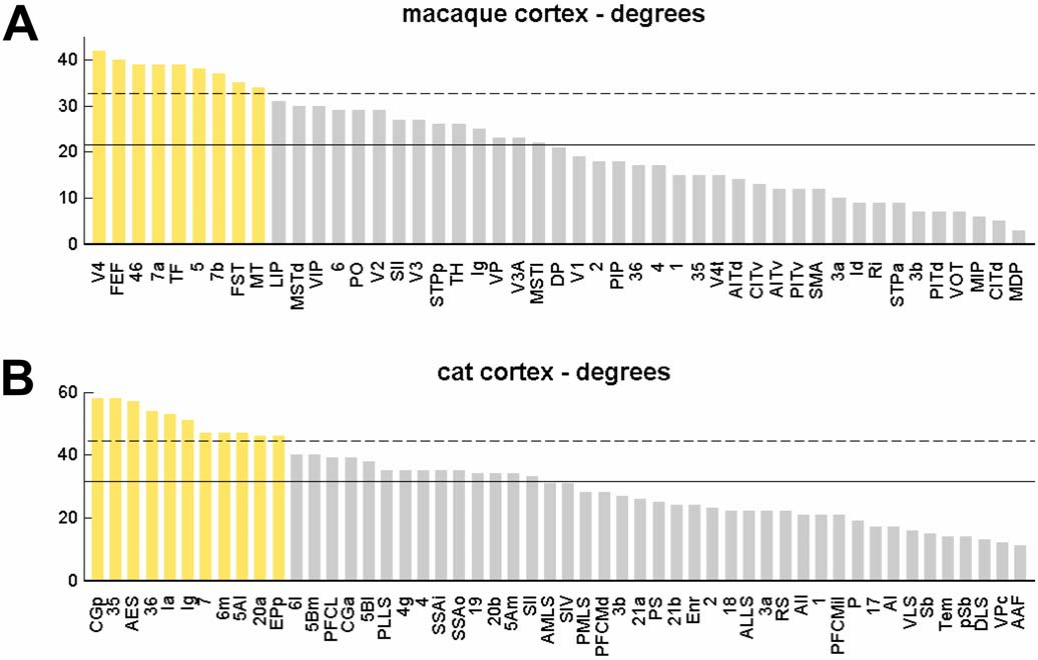
Degree of areas in macaque and cat cortex. The degree of each area of macaque cortex (A) and cat cortex (B) is calculated as the sum over all row and column entries for that area in the matrix of structural connections (Fig. 1). High-degree areas are all areas with a degree greater than the network mean plus one standard deviation. In this, and in all subsequent figures in this paper, these high-degree areas are labeled in yellow.

The connection matrices for macaque and cat cortex were of similar size and density. Both matrices contained a high fraction of reciprocal pathways (0.76 in the macaque cortex, 0.74 in the cat cortex). Vertex degrees for each matrix are shown in Figure 2. In both matrices, degrees varied over a broad range without presenting evidence of a scale-free organization. For the remainder of this paper all areas with a degree that is at least one standard deviation greater than the mean are termed “high-degree areas” (Figure 2). Both networks were fully connected, and the maximal distances (diameter) did not exceed four edges. Average path lengths and clustering coefficients indicated that macaque and cat cortex exhibit small-world attributes, confirming several earlier reports on similar data sets (reviewed in [10]). The degree to which each network resembles a small world can be quantified by the small-world index [34], found to be *σ_sw_* = 1.4551 (±0.0408) for macaque cortex and *σ_sw_* = 1.3153 (±0.0148) for cat cortex (mean and s.d., n = 1000 random networks).

**Figure 3:**
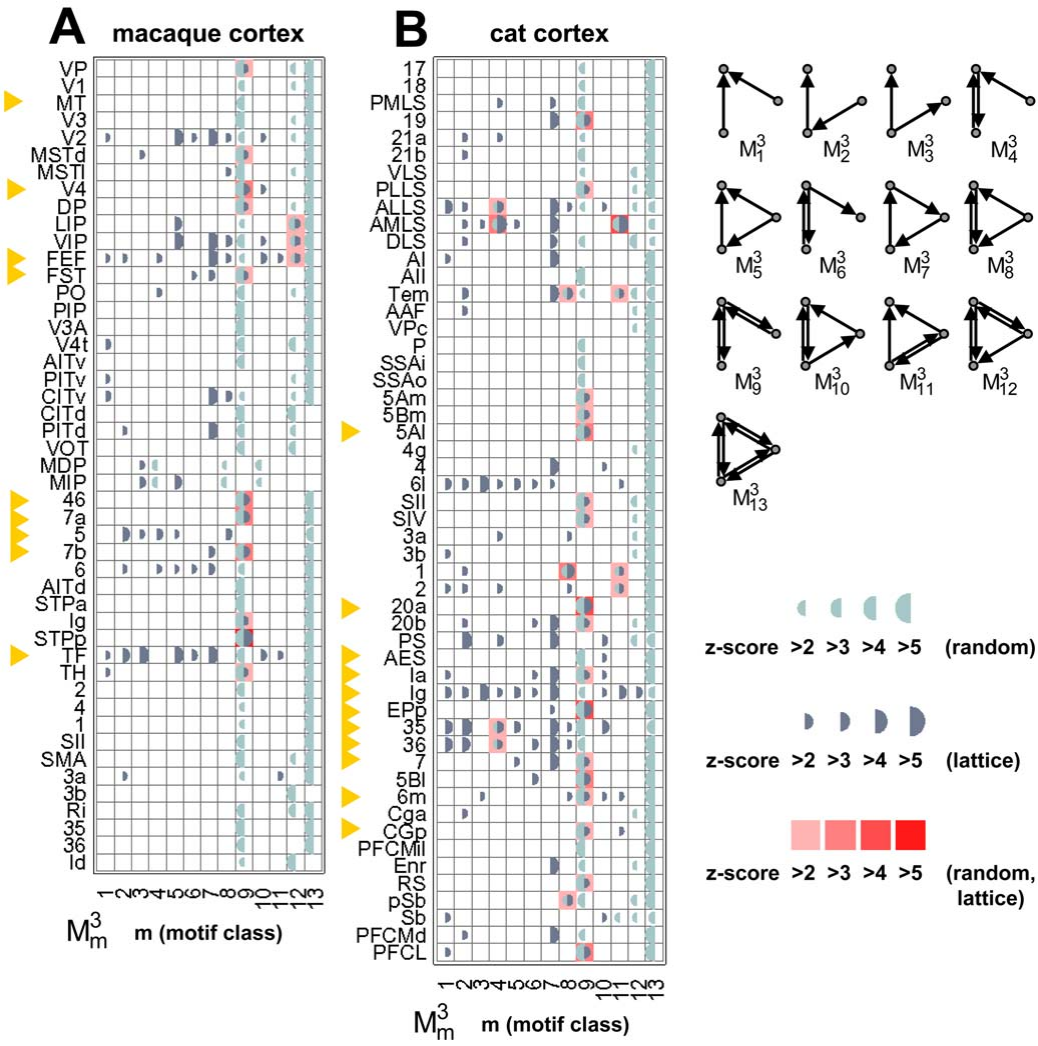
Statistical significance of motif participation for individual brain regions. (A) Macaque cortex. (B) Cat cortex. Each plot shows z-scores (half circles, light blue = relative to random networks, dark blue = relative to lattice networks) for each individual area. Areas with significantly positive z-scores for both comparisons are marked in shades of red (see legend). These areas are marked identically in Fig. 4. High-degree areas are marked by yellow arrows. Motif classes of size M = 3 are shown at the upper right of the plot.

We derived structural motif frequency spectra for motifs of size *M = *3 for both connection matrices (data not shown). Confirming earlier results for similar connection patterns [20], motif spectra for macaque and cat cortex were highly correlated (*r^2^* = 0.88, p<10^−5^) and both data sets exhibited an overabundance of a single motif class, here denoted 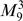 (see Figure 3). Overabundance was assessed by computing z-scores for comparisons to *n = *100 equivalent control networks (random and lattice, see Methods). A motif was considered “significantly increased” if, relative to both random and lattice control networks, its z-scores exceeded *z = *3. We note that lattice controls have near-equal proportions of reciprocal edges as compared to the actual data sets, indicating that a high proportion of reciprocal edges alone does not explain the overabundance of motif 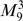. Motif analysis for larger motifs (*M* = 4, *M* = 5) identified several motif classes as significantly increased over both random and lattice controls, including various tilings of motif 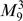 into ring and star patterns (data not shown).

Individual brain regions make specific contributions to the overall motif distribution of the network. Specifically, we sought to identify regions that disproportionately contribute to motif class 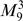 in macaque and cat cortex. To pinpoint locations where aggregations of specific motifs might occur we examined all the participating individual areas in particular structural motifs. The preservation of degree sequences for random and lattice control networks allowed the identification of control vertices that corresponded to those in the real data set, which enabled us to perform statistical comparisons of motif participation on a vertex-by-vertex basis. Figure 3 shows significance profiles for individual brain regions in macaque and cat cortex, revealing that individual brain regions made very different contributions to the global motif frequency spectrum. In macaque and cat cortex the majority of significantly increased contributions involved motif 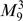. In the macaque, motifs with participation significantly increased from both random and lattice networks were 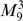 (for 11 vertices) and 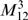 (for 3 vertices), while in the cat significant increases were observed in 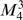 (for 4 vertices), 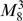 (for 3 vertices), 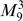 (for 17 vertices), and 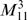 (for 4 vertices). In macaque areas with increased contributions to 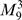 participated in the majority in the dorsal stream of visual processing (MSTd, DP) or in polysensory integration (7a, 7b, STPp, 46, Ig), with the notable exception of areas VP and V4 that are believed to be components of the ventral visual processing stream. Significantly increased motif 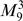 in the cat also occurred among a subset of polysensory regions (PLLS, 20a, EPp, PFCL, Ia, CGp, RS), notably extending also to ‘higher’ motor (area 6 m) and sensory (visual: area 19; somatosensory: SII, SIV) regions.

**Figure 4:**
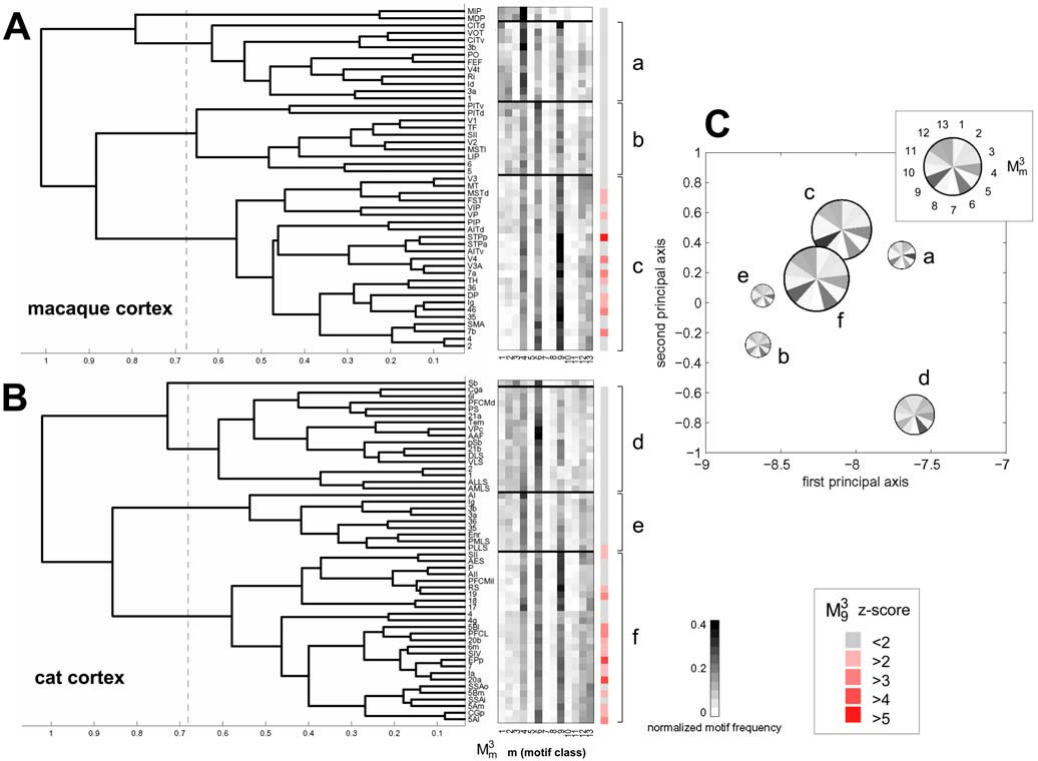
Hierarchical cluster analysis of motif fingerprints for individual brain regions. (A) Dendrogram and clustered motif fingerprints for macaque cortex (top) and cat cortex (bottom). The dendrogram was constructed from the Pearson correlations between all pairs of motif fingerprints (normalized) using a consecutive linking procedure based on farthest inter-cluster distances. This results in a dendrogram with smaller distances for areas with more similar motif fingerprints. Stippled lines mark 2/3-maximal distance, at which cluster boundaries were drawn for subsequent analysis. Motif fingerprints for individual brain regions are arranged vertically by hierarchical cluster distance. Four distinct clusters per network are delineated and clusters with more than 2 members are marked “a”, “b”, “c” for macaque cortex, and “d”, “e”, “f” for cat cortex. (B) The average motif fingerprints for these six clusters are used to perform principal components analysis (PCA); a PCA plot spanning the two largest principal axes is shown. Average motif fingerprints are plotted as segmented circles, with circle size proportional to the number of contributing areas within the cluster, and motif classes represented around the circle (see inset). Note the proximity of several regional clusters with highly similar average motif fingerprints, especially clusters “c” (macaque cortex) and “f” (cat cortex).

Motif fingerprints summarize the participation of individual brain regions in specific motif classes [20]. We derived motif fingerprints for all brain areas of macaque and cat cortex and then performed hierarchical cluster analysis and principal components analysis on these fingerprints to reveal clusters of brain regions with similar motif fingerprints (Figure 4). We found that macaque and cat motif fingerprints formed approximately equal numbers of clusters, and that several of the average motif fingerprints of these clusters shared substantial similarity. The two main clusters for macaque and cat (labeled “c” and “f” in Fig. 4A,B) yielded highly similar average motif fingerprints. Following principal components analysis these two patterns were placed in close proximity (Figure 4). All areas with significantly increased participation for motif 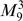 were found within these two clusters (with the exception of area PLLS in cat cortex). The cluster structure observed in cat and macaque cortex did not appear if cluster analysis was carried out on degree-matched random or lattice control networks.

**Figure 5:**
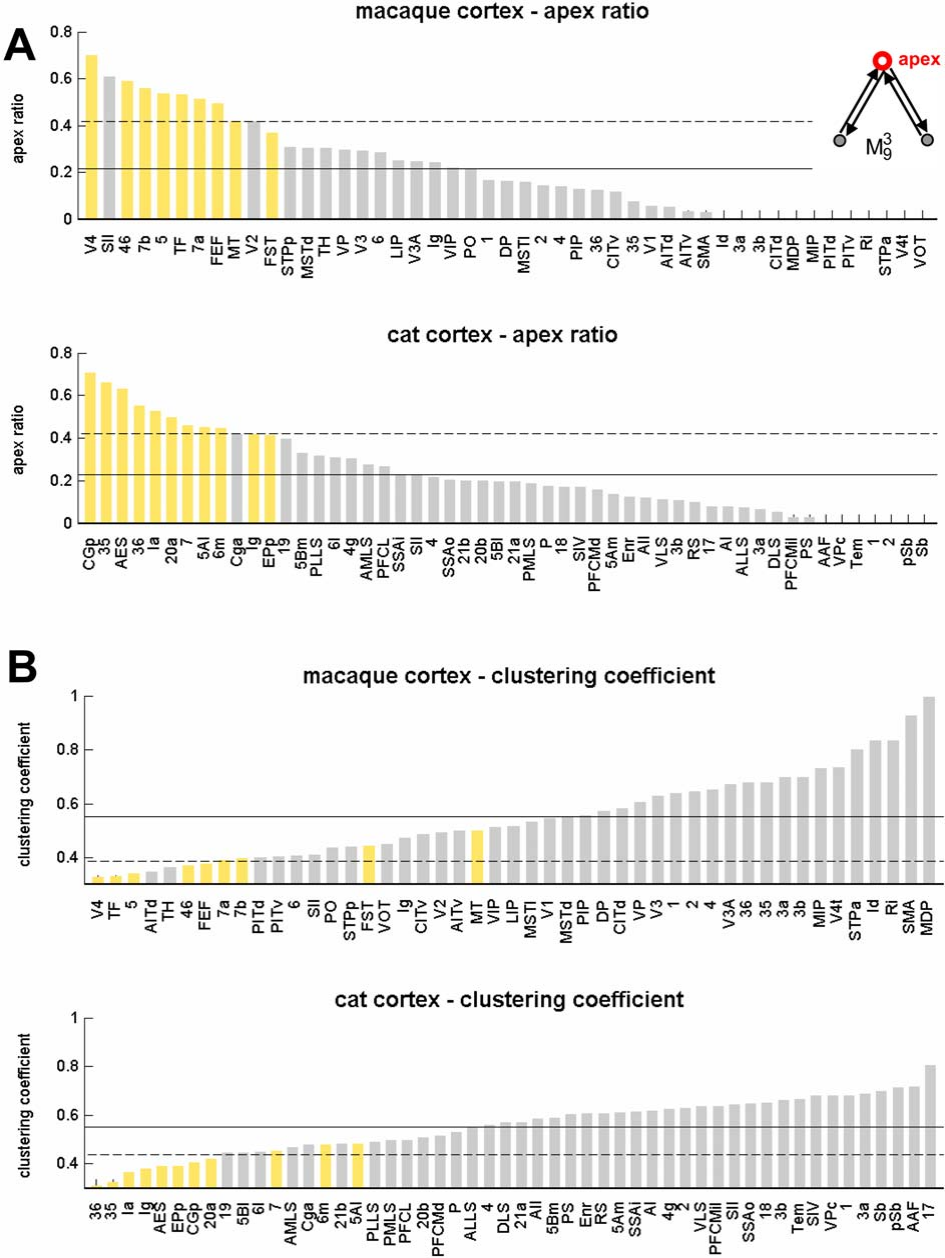
Apex ratio for motif 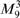 and clustering coefficients in macaque and cat cortex. The apex ratio (A) reflects the incidence of a given brain region at the apex (central node) of all motifs of class 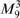 that the region participates in (see inset). Areas are displayed in decreasing order. High-degree areas are displayed with yellow bars, others are displayed with gray bars. Horizontal lines mark the mean apex ratio (solid line) and the mean plus one standard deviation (dashed line). Panel B shows the ranked clustering coefficient for each area of macaque and cat cortex, with high-degree areas once again shown in yellow. Horizontal lines mark the mean clustering coefficient (solid line) and the mean minus one standard deviation (dashed line).

Area V4 participates in 136 instances (out of 721) of motif 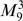, the largest contribution of any area in macaque cortex. In 96 of these instances area V4 is found at the central apex of this motif (Fig. 5A, inset). We define the apex ratio as the fraction of apex locations out of all instances of motif 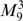, yielding an apex ratio of 0.701 for area V4 (random placement would yield an apex ratio of 1/3). Apex ratios for all areas in macaque and cat cortex are shown in Figure 5A. In both species, all high-degree areas exhibit high apex ratios for motif 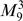. High contributions to motif class 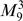 , combined with a high apex ratio, should be associated with low values for the clustering coefficient, as only a relatively small fraction of neighbors are connected with one another. Figure 5B shows that clustering coefficients are indeed found to be below the network mean for all high-degree areas.

**Figure 6:**
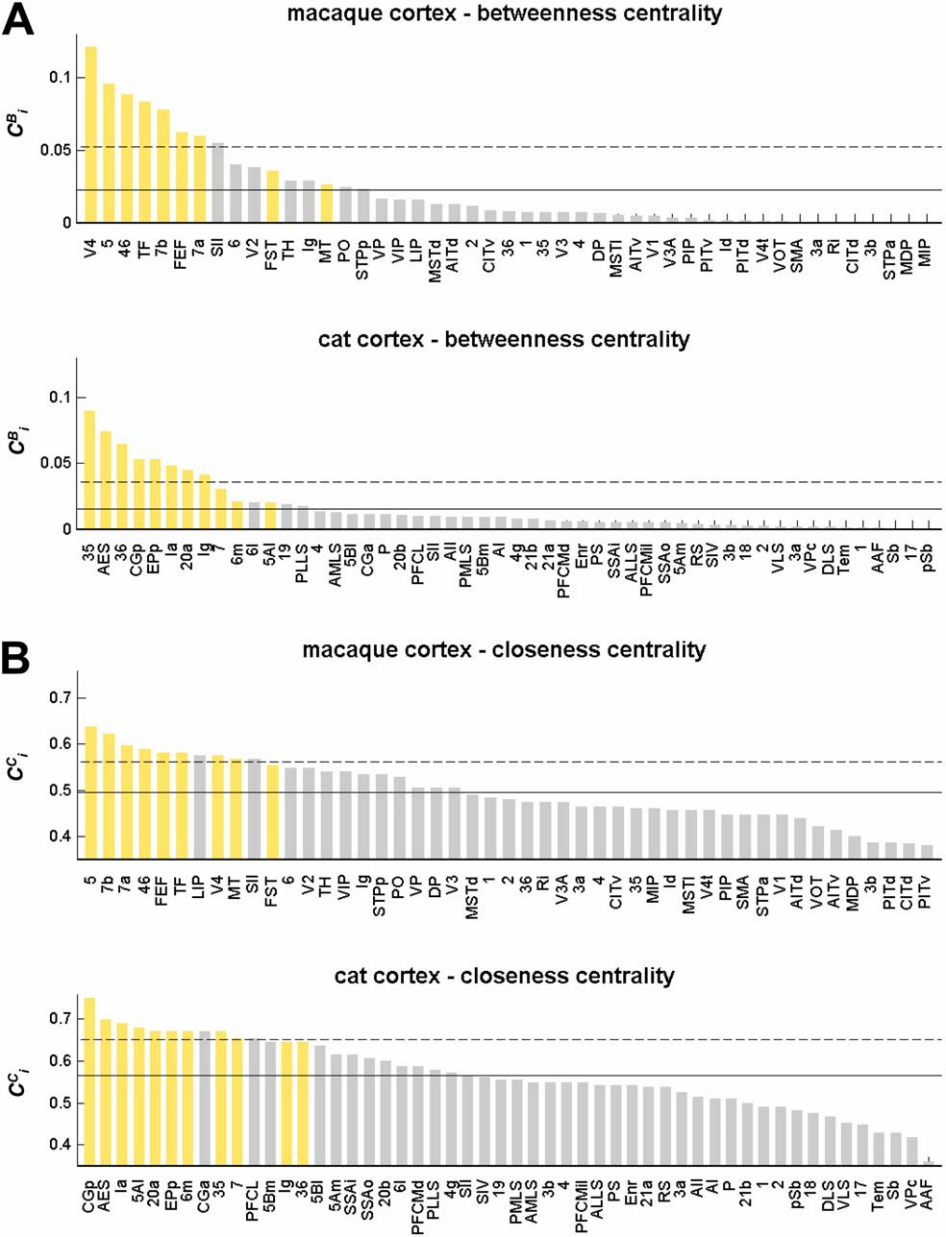
Centrality measures in macaque and cat cortex. (A) Betweenness centrality, calculated as the fraction of all shortest paths traveling through a given vertex (see Methods). (B) Closeness centrality, calculated as the inverse of the average length of the shortest paths linking a given vertex to all others in the network (see Methods). Areas are ranked in decreasing order, with high-degree areas shown in yellow. Horizontal lines mark mean centrality (solid line) and mean plus one standard deviation (dashed line).

Apex ratios and clustering coefficients suggest that brain regions with significantly increased contributions to motif 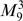 form topological hubs of reciprocal edges, linking many diverse vertices. We might expect that these local waystations have high network centrality. Of the numerous available centrality measures we calculated two: betweenness centrality ([37]; Fig. 6A) and closeness centrality ([38]; Figure 6B). Betweenness centrality captures the degree to which a given brain region participates in the set of shortest paths between any pair of vertices in the network. Closeness centrality captures the average closeness (defined as the inverse of the shortest path length) to all other vertices. In macaque cortex, areas V4, 46, 7a and 7b (previously identified as making significantly increased contributions to motif 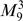, and having high apex ratios as well as low clustering coefficients) are among those with the highest betweenness centrality as well as closeness centrality. In cat cortex, areas CGp, EPp, Ia, and 20a share the same characteristics. Without exception, and in both species, areas with high degree have greater than average centrality.

All of the measures considered so far are interrelated, primarily through the most basic characteristic of each vertex, its degree. As expected, degree and clustering coefficient (r^2^ = 0.44 in macaque, r^2^ = 0.67 in cat) and degree and betweenness (r^2^ = 0.68 in macaque, r^2^ = 0.68 in cat) are moderately cross-correlated. Among motif classes, centrality is on average most strongly correlated with motif 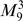 (r^2^ = 0.55 in macaque cortex, r^2^ = 0.67 in cat cortex) while other highly connected motifs (e.g. 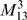) reach comparable levels. Tables 1 and 2 summarize our analysis for all high-degree nodes in macaque and cat cortex. On the basis of several intersecting criteria, we can identify areas V4, FEF, 46, 7a, TF, 5, and 7b as the strongest candidates for hub regions in macaque cortex, while areas CGp, 35, AES, Ia, 20a and EPp are the strongest candidates for hub regions in cat cortex.

**Table 1:** Summary of results for hub identification and hub classification for high-degree areas in macaque cortex.

area name	hub identification						hub classification					
	*k*	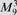	*ap*	*γ*	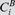	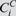	P≥0.3	P<0.3	l≥s	l<s	les+	les–
V4	1	>3	1	1	1	8		•		•		•
FEF	2			7	6	5	•		•		•	
46	3	>3	3	6	3	4	•		•			
7a	4	>3	7		7	3	•		•		•	
TF	5		6	2	4	6	•		•			
5	6		5	3	2	1	•		•			
7b	7	>3	4		5	2	•			•		
FST	8	>2					•			•	•	
MT	9					9		•		•		
SII			2		8	10		•		•		•

**Figure 7:**
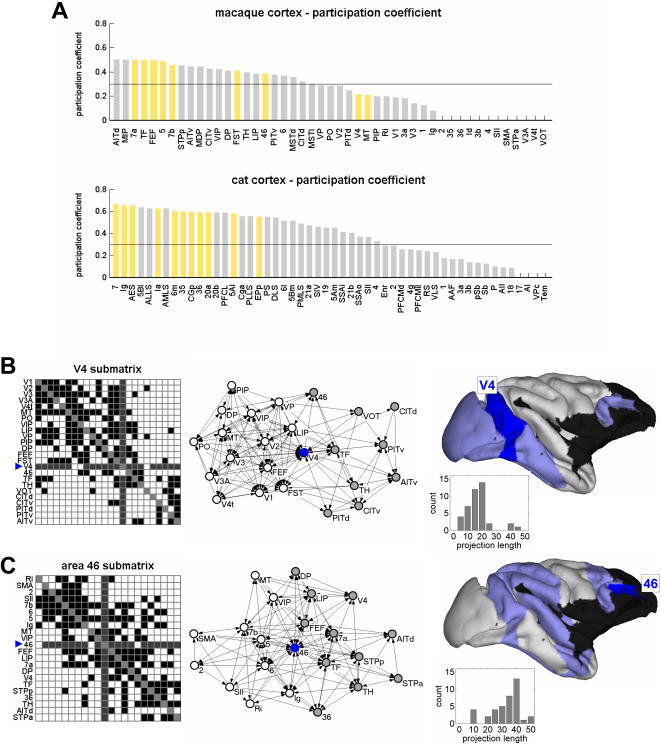
Hub classification. (A) Distribution of participation coefficients (see Methods) for each area of macaque and cat cortex, ranked in decreasing magnitude, with high-degree areas shown in yellow. (B,C) Area V4 and area 46 submatrices and projection length distributions. (B, left) Area V4 submatrix, comprised of the subset of areas and connections of the macaque cortex directly connected to area V4. Areas are arranged such that connections are optimally contracted towards the main diagonal, resulting in two clusters containing mostly dorsal (upper left) and mostly ventral (lower right) areas. V4 afferents and efferents are shaded in dark gray. (B, middle) Graph rendering of the V4 submatrix shows that this subnetwork comprises two component clusters with V4 in a central position. Rendering of the graph was performed in Pajek (http://vlado.fmf.uni-lj.si/pub/networks/pajek/; [69]) using the Kamada-Kawai layout algorithm [70]. V4 is marked by a blue dot, members of cluster 1 (mostly dorsal stream visual areas) are marked in white, and members of cluster 2 (mostly ventral stream visual areas) are marked in gray. (B, right) Surface representation of V4 (shaded in blue) and its direct neighbors (shaded in light blue). Histogram shows the distribution of the connection lengths between area V4 and its immediate neighbors. The mean connection length is 17.09 mm (S.D. = 9.60 mm). (C, left) Area 46 submatrix. (C, middle) Pajek plot for area 46 submatrix. Clusters linked by area 46 appear less segregated than those for area V4 and contain a mixture of visual, sensorimotor and multimodal areas. (C, right) Surface representation of area 46 and its neighbors, and histogram of area 46 connection lengths (mean = 33.41 mm, S.D. = 10.58 mm).

**Figure 8:**
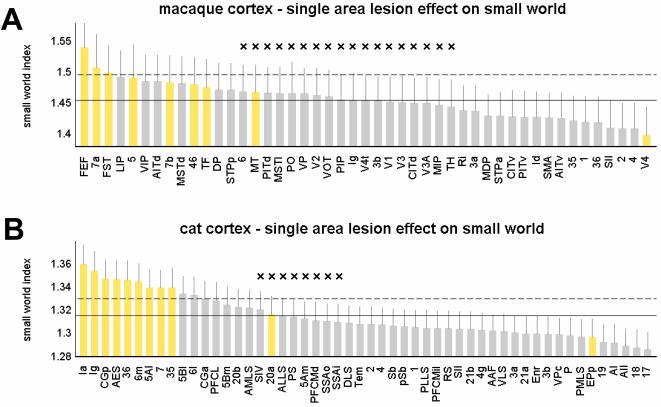
Impact of single area lesion on small-world index. Areas are sorted in decreasing order and high-degree areas are shown in yellow. Horizontal lines mark the mean small-world index of the intact macaque (A) and cat (B) network (solid lines) as well as their respective standard deviations (dashed lines). Error bars show the standard deviations of the distributions of *σ_sw_* after the lesion was made. All distributions for *σ_sw_* were derived from n = 1000 comparisons to degree-matched random networks. Differences at the ends of the spectrum were highly significant (p<10^−16^); non-significant differences are marked with a cross (×).

Measures listed under ‘hub identification”: *k* = degree (Figure 2), 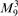 = z-score relative to random and lattice controls (Figure 3), *ap* = apex ratio (Figure 5A), *γ* = clustering coefficient (Figure 5B), 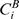 = betweenness centrality (Figure 6A), 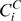 = closeness centrality (Figure 6B). For all measures summarized under hub identification (except motif z-scores), table entries refer to rank within the respective distribution. No rank is given if the measure deviated by less than one standard deviation from the mean. Measures listed under hub classification refer to the participation index P (Fig. 7A), the number of long versus short connections (long connections are defined as having a length greater than the network average of 18 mm), and the direction of the lesion effect on the small-world index (les+and les-, respectively) refer to an increase or a decrease over the small-world index of the unlesioned matrix by more than one standard deviation; see Figure 8.

**Table 2:** Summary of results for hub identification and hub classification for high-degree areas in cat cortex.

area name	hub identification						hub classification					
	*k*	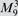	*ap*	*γ*	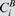	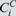	P≥0.3	P<0.3	l>s	l<s	les+	les–
CGp	1	>2	1	7	4	1	•		not available		•	
35	2		2	2	1	9	•				•	
AES	3		3	5	2	2	•				•	
36	4		4	1	3		•				•	
Ia	5	>2	5	3	6	3	•				•	
Ig	6			4	8		•				•	
7	7	>2	7			10	•				•	
6 m	8	>2	9			7	•				•	
5Al	9	>3	8			4	•				•	
20a	10	>4	6	8	7	5	•					
EPp	11	>4		6	5	6	•					•

All symbols are as for Table 1.

Once network hubs have been identified, hubs may be classified on the basis of whether their connections are distributed mostly within or mostly between network modules [28], [42]. Hubs may also be classified on the basis of their spatial embedding, e.g. the distribution of the metric lengths of their projections [22]. We pursued both approaches to hub classification. We applied a spectral community detection algorithm [41] to identify modules within macaque and cat cortex. We extracted optimal community structures with 2 (macaque) and 3 modules (cat). Figure 7A plots the participation coefficient *P*, which expresses, for each area, the balance between connections that are made within and between modules. Following a previously published classification scheme [28], [42], we denote high-degree areas with P>0.3 as connector hubs, while high-degree areas with P<0.3 are denoted as provincial hubs (Table 1,2). In macaque cortex, the majority of hubs are connectors, while areas V4 and MT, as well as the less highly connected yet highly central area SII are classified as provincial hubs. In cat cortex, all high-degree areas are classified as connector hubs. The absence of clearly defined provincial hubs may point to a difference in the structural organization of network modules in the two species.

To visualize the structural embedding of a provincial and a connector hub, we plotted two submatrices of macaque visual cortex, comprising area V4 (a provincial hub) and area 46 (a connector hub) together with their immediate topological neighbors (Figure 7B,C). The V4 submatrix (Figure 7B, left) and a corresponding cortical surface representation (Figure 7B, right) indicates that virtually all of V4's neighbors are located within visual cortex, with most of V4's inter-regional connections spanning relatively short distances (17.09 mm±9.60 mm s.d.). Of its 42 connections with other areas, 23 are shorter than the network's mean connection length of 18 mm (Table 1). The graph structure of the V4 submatrix (Figure 7B, middle) suggests that V4 mediates information flow between two groups of areas, one belonging predominantly to the dorsal visual stream (with the exception of area VP) and the other belonging to the ventral visual stream (with the exception of area 46, a connector hub). In contrast, corresponding plots for area 46 (Figure 7C) reveal that this area maintains a more diverse set of projections, including visual, somatosensory and motor regions. Many of the connections of area 46 were found to span long distances (33.41 mm±10.58 mm s.d.; significantly different from those of V4, p<0.0001, with 35 of its 39 connections longer than 18 mm), although we note that some short projections are likely missing because other prefrontal regions were not included in the connection matrix. Connector hubs are very highly interconnected amongst themselves, forming “hub complexes” with a connection density of 0.81 (macaque) and 0.85 (cat). For comparison, submatrices of areas with identical degrees sampled from randomized control networks have connection densities of 0.69±0.06 (n = 1000, p<0.02) in macaque and 0.76±0.03 (n = 1000, p<0.01) in cat cortex.

Lesions of hubs may be expected to have unusually large consequences on information flow and communication within the remaining network. Such consequences may structurally be assessed by plotting changes in the network's path length and clustering coefficient following the lesion. Our analysis shows that the effect of lesioning a single area on the network's small-world index is just as likely to be positive as negative. Figure 8 summarizes the impact of single area lesions on the small-world index for macaque cortex and cat cortex. In macaque cortex, lesions of connector hubs such as FEF, 46, 7a, 7b (or more generally, areas with high participation coefficient) resulted in large increases in the small-world index relative to the unlesioned network. This effect is due to an increase in cluster distance (expressed in an increase in path length) as well as an increase in their segregation from each other (expressed in an even greater increase in clustering). In contrast, lesions of provincial hubs (e.g. area V4) or more generally of areas with low participation coefficient (e.g. area SII) resulted in decreases of the small-world index. This decrease is due to a decrease in clustering accompanied by a smaller effect (an increase or a decrease) in the path length. Similar patterns were found in cat cortex, with lesions of high-degree connector hubs such as Ia and CGp resulting in a higher small-world index, while lesioning of areas with lower participation coefficient had the opposite effect. In both, macaque and cat cortex, distributions of small-world lesion effects and participation coefficients over all areas are highly and significantly correlated (see Text S1). These results indicate that lesions of hub regions belonging to different classes may have differential effects on the small-world structure of the remaining network.

## Discussion

In this paper, we have examined the structural contributions of individual cortical areas to large-scale cortical networks of the cat and the macaque monkey. Earlier studies have provided evidence for a high degree of clustering and short path lengths within cortical networks of cat and macaque [7], [8], [10]. Such small-world networks likely contain a subset of areas that act as hubs or bridges which should be identifiable based on structural attributes that relate to their functional roles. Using multiple structural measures we identified sets of such areas in both cat and macaque cortex and demonstrated marked similarities across brain functional systems and species. High degree regions made significantly increased contributions to structural motif 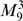 and also tended to have high centrality. We separated hub regions into provincial and connector classes, and showed that lesions of the two types of hubs had opposite effects on the small-world organization of the remaining network.

To reliably identify hubs in brain networks, we used multiple structural measures, including vertex degree, motif participation and betweenness and closeness centrality. While hubs are most often identified solely on the basis of their high degree, the relationship of degree to other aspects of their structural embedding is less well understood. While clearly interrelated, each of the measures we apply in this study captures a distinct way in which an area participates in the structure of the whole network. In our combined analysis of motif contributions and centrality we noted that, for high-degree nodes, motif 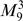 appears to be associated with high centrality. Many high-degree nodes with high 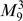 contributions and centrality correspond to brain regions that are functionally classified as “polysensory”, or “multimodal association areas”, including several parietal and dorsolateral prefrontal cortical regions, in addition to posterior cingulate cortex and the insula. The remaining, apparently unimodal, sensory regions are not found at lower hierarchical levels but may be classified as ‘higher’ areas (e.g. area 19 in visual cortex and area 5 in somatosensory cortex). It is well known that these areas receive direct projections from other sensory modalities, and functional responses to crossmodal stimuli have been demonstrated [43]–[45]. Furthermore, the correspondence we find between structural centrality and polymodality is consistent with recent human fMRI studies [23] which showed that association cortices have the highest regional efficiency (or, equivalently, the highest closeness centrality) within brain functional networks, regardless of the age of subjects.

Following the notion that function is an expression of structural connectivity suggested by Passingham et al. [18], areas become polysensory or multimodal *because* of the way in which they participate and are embedded in the larger network. Contribution analysis as employed in this study may provide a method for the classification of brain regions that is complementary to the more commonly used categories (sensory/motor, unimodal/multimodal, primary/secondary), and which is based on an objective quantification of inter-regional connectivity.

We find a strong link between increased contribution to motif 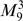, high centrality, and the impact of lesions on global network measures that are thought to relate to information flow and integration. This link makes predictions about the role of 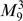 and centrality in robustness of other brain networks and suggests that network recovery might involve the substitution of vertices with high 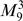 and centrality, to ensure high transmission while maintaining segregation. Previous studies [46], [47], investigated the vulnerability of large-scale cortical networks by analyzing the structural impact of deleting individual edges or vertices. The frequency with which an edge occurred in all shortest paths (a measure related to “edge betweenness”; [46]) was found to be highly correlated with vulnerability.

Hubs may be classified as provincial or connectors [28], with provincial hubs linking vertices primarily within a single cluster, and with connector hubs linking multiple clusters to one another. Building on this topological classification scheme, recent functional connectivity studies [22] have suggested that brain regions with high centrality may either link to other regions within a more local neighborhood (i.e. likely forming a single functional cluster) or interconnect to regions over longer distances (i.e. likely members of different functional clusters). A recent simulation study of functional connectivity in macaque visual cortex noted that some functional hub regions appeared to integrate information within a single cluster, e.g. visual (V4) or sensorimotor (SII), while other regions (46, 7a, 7b) appeared to connect visual and sensorimotor clusters to one another [24]. The present paper allows us to differentiate these two types of functional hubs on the basis of their structural embedding. Figure 7B,C shows the distribution of connection lengths for a provincial hub (area V4) and a connector hub (area 46), suggesting that these hub types map onto the distinction proposed for functional connectivity [22]. Connector hubs constitute a large proportion of the long-range intra-hemispheric pathways within the cortical system, underscoring their potential importance for minimizing the number of processing steps [15].

We find that the deletion of provincial hubs and the deletion of connector hubs have distinctly different effects on the small-world structure of the remaining network (Figure 8). Deletion of connector hubs disconnects functional clusters, rendering them at the same time more remote and more distinct, thus resulting in a relative increase in the small-world index. Instead, deletion of provincial hubs disturbs the functional integration of the cluster to which they belong, which then renders the remaining network less segregated.

Our analysis suggests a special status for area V4 in macaque visual cortex. We find that V4 makes the largest single contribution to motif 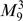 in its parent network, exhibits the highest apex ratio and betweenness centrality, and acting as a provincial hub its deletion diminishes the small-world architecture of the remaining network. Do these structural features reflect known functional characteristics of area V4? Numerous physiological studies of monkey V4 have suggested that V4 is involved in a broad range of complex visual functions, not limited to one visual modality, including color, texture and form vision [48]. Lesions of V4 result in deficits in visual tasks that do not rely on a single visual modality [48], including in visual recognition [49] and in attentional processing [50]. The abundance of functional evidence suggesting a central role for V4 in the integration of information from different components of the visual system is consistent with its structural embedding as reported in this study.

Macaque area 46, which was identified here as a paradigmatic connector hub, and which has previously been found to serve a connecting functional role in large-scale cortical modeling [24], is a key region receiving polysensory inputs from posterior cortex, integrating external information with internal goals, and keeping this information online over time for action [51]–[53]. This complex function underlies what is variously described as, for example, working memory, spatial (dorsal) or object (ventral) cognition, selective attention or the “central executive” [51], [53]–[55] and the prediction of future reward [56]. Lesions in this cortical region lead to a typical dorsolateral syndrome with the hallmark of a lack of drive and awareness [53]. Area 46 also has an unusually large number of connections to spatially distant parietal regions. Assuming that axonal conduction delays increase with projection length, this suggests that area 46 has to process information on a wider variety of time scales than most other brain regions. This structural observation is in accord with the finding that neurons in this area perform ‘active maintenance’ during delay tasks [55]. The frontal eye fields (FEF), which also send and receive many long-range pathways, have also been found to maintain and transmit delayed signals [57]. One of the most prominent differences in the connection profiles of prefrontal area 46 and the FEF is that the latter sends efferents to areas V2, V3, V3A and V4t while the former communicates only with area V4. Thus, while areas 46 and FEF are both connector hubs, the function of FEF is more tightly related to the visual modality, as was noted by previous structural analyses [58].

The use of data sets from two different mammalian species (macaque monkey and cat) invites comparisons between these structures in terms of a broad range of graph theoretical measures, including small-world attributes, motif composition and centrality. Cross-species comparisons of brain connectivity patterns are made difficult by the fact that only very few comprehensive data sets collated from anatomical tract tracing studies are currently available. Other potential problems include differences in size and density of connection matrices, the use of different parcellation schemes, data sources, anatomical tracing methods, spatial resolution, inclusion or lack of thalamic regions, and uncertain regional homologies. Despite these problems we suggest that graph-theoretical descriptors have the potential to provide significant new insights into patterns of brain evolution [59] going beyond the consideration of brain size, average connectedness, or wiring length. Future anatomical data bases enlarging the range of species may be constructed from genetic markers [60], selectively activated viral tracers [61], novel optical imaging techniques [62] or from diffusion imaging of brain tissue [63], [64]. These data sets would provide “connectomes” [65] or “projectomes” [66] at high spatial resolution and allow much more fine-grained analyses of complex brain networks, and would thus provide new insights into functionally relevant patterns that are conserved or elaborated during brain evolution.

Our aim was to link aspects of functional specificity and performance of brain regions, in particular of network hubs, to their structural embedding within cortical networks. Many extensions and refinements of this work are possible. These include the analysis of data on strengths or density of connections, the investigation of refined partitioning schemes in different cortical functional systems, the spatial embedding of brain regions and pathways (e.g. [15]), the consideration of hierarchical network measures (e.g. [67]) and application of the graph theory framework to functional connectivity data (e.g. [21], [22], [24]). With the arrival of new structural imaging methods, it is now feasible to apply network analysis to human anatomical data [64], [68], and thereby to further our understanding of how human brain anatomy relates to cognition.

## Supporting Information

Text S1 (0.49 MB DOC)
