# Supplementary material for: Identification and Classification of Hubs in Brain Networks
Source: PLoS One. 2007 Oct 17;2(10):e1049. doi: 10.1371/journal.pone.0001049 (PMC2013941; doi:10.1371/journal.pone.0001049)

**Identification and Classification of Hubs in Brain Networks**

Olaf Sporns,^1^ Christopher J. Honey,^1^ Rolf Kötter^2^

^1^ Department of Psychological and Brain Sciences and Program in Cognitive Science, Indiana University, Bloomington, Indiana 47405, USA.

^2^ Department of Cognitive Neuroscience, Section Neurophysiology & Neuroinformatics, Radboud University Medical Center, 6500 HB Nijmegen, The Netherlands; C.&O. Vogt Brain Research Institute and Institute of Anatomy II, Heinrich Heine University, Moorenstr. 5, D-40225 Düsseldorf, Germany.

**SUPPLEMENTARY MATERIAL**

**Connection Data Sets**

The four connection data sets can be downloaded at the links provided below. Files are in Matlab format, with binary connection matrices (‘CIJ’) and names of brain regions (‘Names’), in the ordering scheme shown in Figure 2 of the manuscript.

<http://www.indiana.edu/~cortex/macaque_cortex.mat>

<http://www.indiana.edu/~cortex/cat_cortex.mat>

**Abbreviations of Brain Regions**

**Macaque Cortex (visual areas after Felleman and Van Essen, 1991)**

VP Ventral Posterior

V1 Visual area 1

MT Middle temporal

V3 Visual area 3

V2 Visual area 2

MSTd Medial superior temporal (dorsal)

MSTl Medial superior temporal (lateral)

V4 Visual area 4

DP Dorsal preluneate

LIP Lateral intraparietal

VIP Ventral intraparietal

FEF Frontal eye field

FST Floor of superior temporal

PO Parieto-occipital

PIP Posterior intraparietal

V3A Visual area V3A

V4t V4 transitional

AITv Anterior inferotemporal (ventral)

PITv Posterior inferotemporal (ventral)

CITv Central inferotemporal (ventral)

CITd Central inferotemporal (dorsal)

PITd Posterior inferotemporal (dorsal)

VOT Ventral occipitotemporal

MDP Medial dorsal parietal

MIP Medial intraparietal

46 Area 46

7a Area 7a

5 Area 5

7b Area 7b

6 Area 6

AITd Anterior inferotemporal (dorsal)

STPa Superior temporal polysensory (anterior)

Ig Insular cortex (granular)

STPp Superior temporal polysensory (posterior)

TF TF

TH TH

2 Area 2

4 Area 4

1 Area 1

SII Secondary somatosensory area

SMA Supplemental motor area

3a Area 3a

3b Area 3b

Ri Retroinsular cortex

35 Area 35

36 Area 36

Id Insular cortex

**Cat Cortex (after Scannell et al., 1999)**

17 Area 17

18 Area 18

PMLS Posteromedial lateral suprasylvian area

19 Area 19

21a Area 21a

21b Area 21b

VLS Ventrolateral suprasylvian area

PLLS Posterolateral lateral suprasylvian area

ALLS Anterolateral lateral suprasylvian area

AMLS Anteromedial lateral surpasylvian area

DLS Dorsolateral suprasylvian area

AI Primary auditory field

AII Secondary auditory field

Tem Temporal auditory field

AAF Anterior auditory field

VPc Ventroposterior auditory field

P Posterior auditory field

SSAi Inner (deep) suprasylvian sulcal region of area 5

SSAo Outer suprasylvian sulcal region of area 5

5Am Medial area 5A

5Bm Medial area 5B

5Al Lateral area 5A

4g Area 4γ

4 Areas 4f, 4sf and 4d

6l Lateral area 6

SII Second somatosensory area

SIV Fourth somatosensory area

3a Area 3a

3b Area 3b

1 Area 1

2 Area 2

20a Area 20a

20b Area 20b

PS Posterior suprasylvian area

AES Anterior ectosylvian sulcus

Ia Agranular insula

Ig Granular insula

EPp Posterior part of the posterior ectosylvian gyrus

35 Area 35 of the perirhinal cortex

36 Area 36 of the perirhinal cortex

7 Area 7

5Bl Lateral area 5B

6m Medial area 6

CGa Anterior cingulate cortex

CGp Posterior cingulate cortex

PFCMil Infralimbic medial prefrontal cortex

Enr Entorhinal cortex

RS Retrosplenial cortex

pSb Presubiculum, parasubiculum and postsubicular cortex

Sb Subiculum

PFCMd Dorsal medial prefrontal cortex

PFCL Lateral prefrontal cortex

**Relation of Small-World Lesion Effect and Participation Coefficient**

Fig. 1SI shows a scatterplot of the magnitude of the small-world lesion effect (data from Fig. 8) and the lesioned area’s participation coefficient (data from Fig. 7A). High-degree areas are marked in yellow.


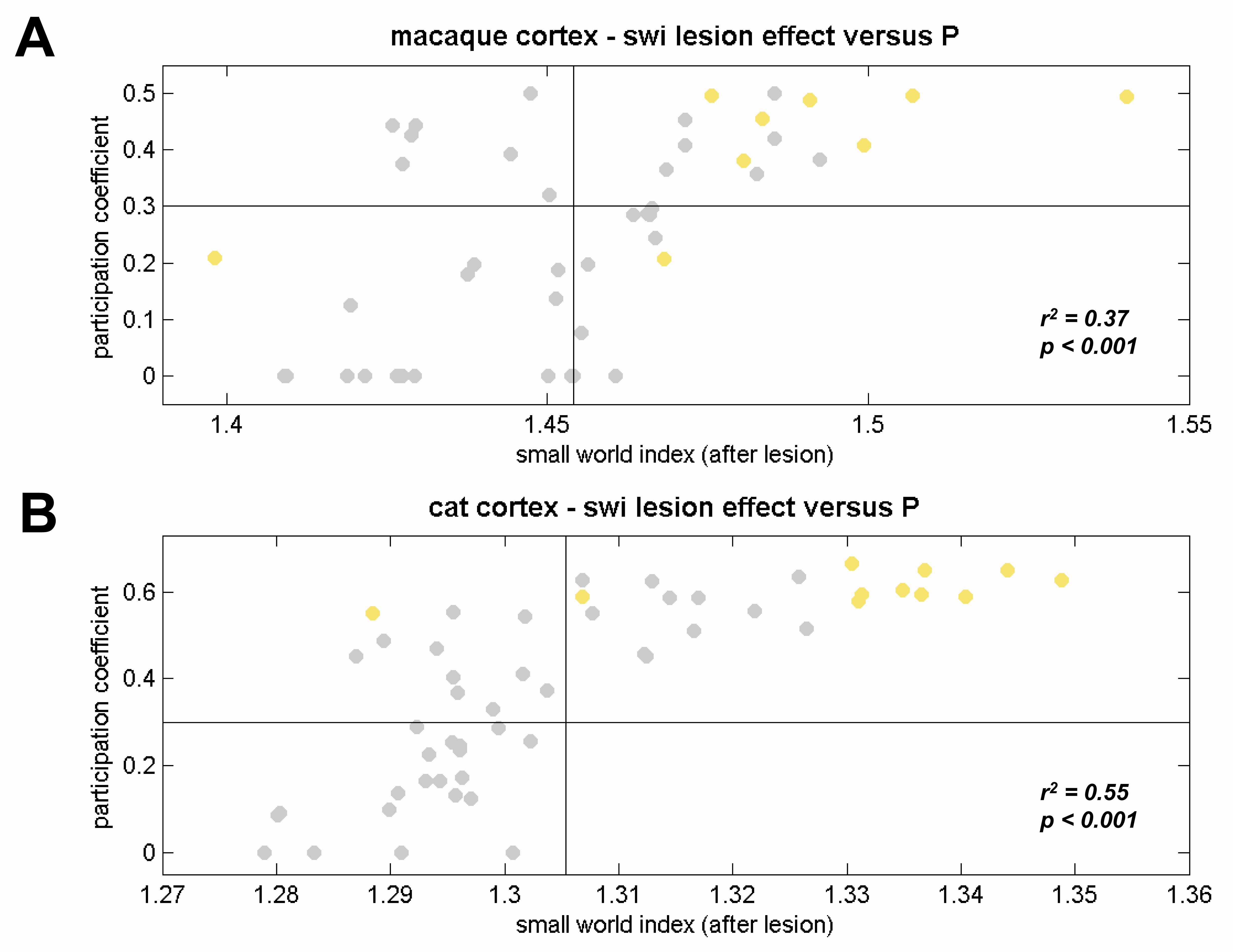

Supplement: Text S1 — (0.49 MB DOC) [file pone.0001049.s001.doc]
